# Influence of working length and anatomical complexities on the apical root canal filling: a nano-CT study

**DOI:** 10.1590/0103-6440202204576

**Published:** 2022-06-24

**Authors:** Natalia Siqueira Lobo, Reinhilde Jacobs, Karla de Faria Vasconcelos, Victor Aquino Wanderley, Bernardo Camargo dos Santos, Marina Angélica Marciano, Alexandre Augusto Zaia

**Affiliations:** 1 Department of Restorative Dentistry, Endodontic Division, Piracicaba Dental School, State University of Campinas, Piracicaba, São Paulo, Brazil.; 2 OMFS IMPATH research group, Department of Imaging and Pathology, Faculty of Medicine, University of Leuven and Oral & Maxillofacial Surgery, Leuven, Belgium.; 3 Department of Dental Medicine, Karolinska Institutet, Stockholm, Sweden.; 4 Department of Oral Diagnosis, Division of Oral Radiology, Piracicaba Dental School, State University of Campinas, Piracicaba, São Paulo, Brazil.; 5 Department of Nuclear Engineering, Federal University of Rio de Janeiro, Rio de Janeiro, Brasil.

**Keywords:** Endodontics, root canal therapy, nanostructures, root canal obturation

## Abstract

The present study aimed to compare the filled volume by gutta-percha and sealer in the apical region of the main canal and ramifications, after instrumentation at two different working lengths using nano-computed tomography (nano-CT). Twenty-two premolars with apical ramifications were selected after micro-computed tomography evaluation and were randomly divided into groups for further endodontic instrumentation at two different working lengths: G1 - Root canals shaped 1 mm short of the apical foramen (n=11), and G2 - Root canals shaped at the apical foramen (n=11). After completing root treatment, nano-CT images were acquired, and the filled volume by gutta-percha and sealer in the main canal apical 0-4 mm and 0-1 mm ranges, and apical ramifications were objectively measured by an operator specialized in both radiology and endodontics, blinded for both groups. The Mann-Whitney test was applied to compare both groups regarding the filling of the main canal apical ranges and apical ramifications with a significance level of 5% (α ≤ 0.05). It was observed that root canals shaped at the apical foramen had a larger volume of the main canal filled than root canals shaped 1 mm short of the apical foramen, at both apical ranges (0-4 and 0-1 mm) (p<0.05). Regarding the filling of the apical ramifications, there was no significant difference between groups (p>0.05). In conclusion, the root canals shaped at apical foramen exhibited increased filling volume of the main canal in the apical region. However, neither of both working lengths influenced filling of the apical ramifications.

## Resumo

O presente estudo teve como objetivo comparar o volume preenchido por guta-percha e cimento na região apical do canal principal e ramificações, após instrumentação em dois comprimentos de trabalho diferentes, por meio de nano tomografia computadorizada (nano-TC). Vinte e dois pré-molares com ramificações apicais foram selecionados após avaliação por micro-tomografia computadorizada e foram divididos aleatoriamente em grupos para posterior instrumentação endodôntica em dois comprimentos de trabalho diferentes: G1 - Canais radiculares instrumentados 1 mm aquém do forame apical (n = 11) e G2 - Canais radiculares instrumentados até o forame apical (n = 11). Após a obturação dos canais radiculares, imagens de nano-CT foram adquiridas, e o volume preenchido por guta-percha e cimento nas faixas apicais de 0-4 mm e 0-1 mm do canal principal, e ramificações apicais, foram avaliadas objetivamente por um especialista em radiologia e endodontia, cego para ambos os grupos. O teste de Mann-Whitney foi aplicado para comparar os dois grupos quanto ao preenchimento das faixas apicais do canal principal e ramificações com nível de significância de 5% (α ≤ 0,05). Observou-se que canais radiculares instrumentados até o forame apical apresentaram maior volume do canal principal preenchido do que canais radiculares instrumentados 1 mm aquém do forame apical, em ambas as faixas apicais (0-4 e 0-1 mm) (p <0,05) Em relação ao preenchimento das ramificações apicais, não houve diferença significativa entre os grupos (p> 0,05). Em conclusão, os canais radiculares instrumentados até o forame apical mostraram um maior volume de preenchimento na região apical do canal principal. No entanto, os dois diferentes comprimentos de trabalho não influenciaram o preenchimento das ramificações apicais.

## Introduction

Complete filling of the cleaned root canal can prevent the development of bacterial infection, influencing the prognosis of endodontic treatment ^1^. Because gutta-percha cannot reach the entire root canal system, the sealer must fill the remaining empty spaces. However, the high prevalence of irregularities and ramifications in the apical region may represent a challenge for the long-term success of endodontic treatment ^2^.

Many clinicians and researchers recognize the importance of cleaning and shaping the root canal space, however, there is no consensus on the ideal extent of the apical limit ^3^ ^,^ ^4^. Some clinical researchers advocate instrumentation of the entire root canal for more favorable healing of the periapical tissues ^5^ ^,^ ^6^; yet, others define instrumentation length at the apical constriction (0.5-2 mm short of radiographic apex) ^3^ ^,^ ^7^.

To date, periapical radiography and cone-beam computed tomography (CBCT) are clinically used to assess the root canal anatomy and to evaluate the filling quality of treatment ^8^. In addition, destructive methods such as clearing technique, scanning electron microscopy, and hard tissue histology are also available for the study of root canals ^9^ ^,^ ^10^. However, their results only provide two-dimensional features and are not completely representative of a complex three-dimensional (3D) structure. High-resolution imaging by microcomputed tomography (micro-CT) and, more recently, nano-computed tomography (nano-CT) can be used for research purposes, yet not for clinical applications considering the high radiation doses and limited field of view. These techniques have been frequently used in endodontic research considering their non-destructive nature and high-resolution 3D images, allowing accurate assessment of structures that normally cannot be clinically assessed ^11^ ^,^ ^12^. Root canal ramifications are a typical example of tiny structures that most of the time are not seen on periapical radiography and CBCT images, but their presence could lead to therapy resistance or eventual failure ^13^.

The study hypothesis is that different instrumentation lengths may influence the filling quality of endodontic treatment. However, yet and until now, no assessment of different working length strategies on filling up the apical main canal and ramifications using 3D high-resolution image modality was noted. Hence, the aim of this study is to compare the filled volume by gutta-percha and sealer in the apical region of the main canal and ramifications, after instrumentation at two different working lengths using nano-computed tomography (nano-CT).

## Material and Methods

The present study was performed after approval by the University Ethics Committee (CAAE 67465617.8.0000.5418).

### Study sample

The study sample was initially composed of 56 extracted human premolars, in accordance with previously published material including sampling power ^11^ ^,^ ^12^ ^,^ ^14^. Teeth were disinfected in 2% glutaraldehyde solution for two hours then scaling was performed to remove dental calculus and remnants of soft tissue. The teeth were kept hydrated in distilled water at room temperature for 1 month before root canal preparation. Crowns were cut at the cementum-enamel junction level using a metallographic precision cutter (IsoMet 1000, Buehler, Lake Bluff, United States of America) and diamond disk. After clinical inspection, Micro-CT images were obtained using a SkyScan 1174 device (Bruker, Kontich, Belgium), operating at 50 kV and 800 µA, with a 0.5 mm aluminum filter, 360° rotation, 0.5° rotation step and isotropic voxel size of 19.6 µm. Micro-CT images were evaluated in consensus by an endodontist and an oral radiologist following the exclusion criteria: teeth with more than one main canal, teeth without root canal ramifications, teeth with more than 3 apical ramifications, presence of endodontic treatment, root dilaceration, root resorption, root fracture, supernumerary roots, obliterated root canals, and pulp calcifications. After exclusion, twenty-two single-rooted premolars with similar volumes of root canals and with apical ramifications were included in the present study.

### Endodontic treatment of the sample

The sample was randomly divided into two equal groups (n=11) according to the different working lengths: G1 - root canals shaped 1 mm short of the apical foramen and the gutta-percha cone was limited at the apical stop (defined as 1 mm short of the apical foramen); G2 - root canals shaped at the apical foramen with the gutta-percha cone stopping 1 mm short of the apical foramen by friction within the dentin walls. The working length was determined by inserting a size number 10 K-file (Maillefer, Ballaiges, Switzerland) until it reached the apical foramen. Instrumen tation was performed using endo-motor VDW silver (VDW GmbH, Munich, Germany) and Reciproc® series of instruments (VDW GmbH) using a crown-down technique; the finishing file was size 40/.06. Throughout the instrumentation steps, the root canal was irrigated with 3mL of distilled water followed by chlorhexidine gel 2% (Biodinâmica, Ibiporã, Brazil). In G1 patency was maintained with a size number 10 k-file after each instrument. The smear layer was removed with 17% EDTA (Biodinâmica) for 1 minute, followed by a final rinse with 3mL of distilled water. Finally, the root canal was dried up with paper points (VDW GmbH) 1 mm short of the apical foramen and Endomethasone (Septodont, St. Maur, France) was prepared in accordance with the manufacturer’s instructions. The single-cone technique was performed using a medium non-standardized master gutta-percha point (Dentsply Maillefer, Ballaigues, Switzerland) calibrated to adapt at the established apical level. The cone was coated with sealer and inserted into the canal until it reached its final position. Then, exceeding material was seared off and condensed with a plugger. A single operator performed all the clinical procedures in both groups prior to scanning the roots were stored at 37°C and in 100% humidity for 10 days to ensure that the sealer was set.

### Imaging acquisition

Nano-CT images were obtained by using Phoenix NanoTom S (GE Sensing & Inspection Technologies GmbH, Wunstorf, Germany) operating at 70 kV, 200 μA, voxel size of 3 μm, 360˚ of rotation, 500 ms exposure, 14 W of power, mode 0 for tube operation and with a 0.5 mm Al filter using the software Phoenix datos | x 3D version 2.6 (GE Sensing & Inspection Technologies GmbH, Wunstorf, Germany). Mean scanning time was 20 min per tooth.

### Image analysis

After scanning, Phoenix datos | x 3D version 2.6 software (GE Sensing & Inspection Technologies GmbH) was used to reconstruct the nano-CT images. All the images were objectively evaluated by one operator specialist in radiology and endodontics, blinded for the different groups.

**Figure 1:**
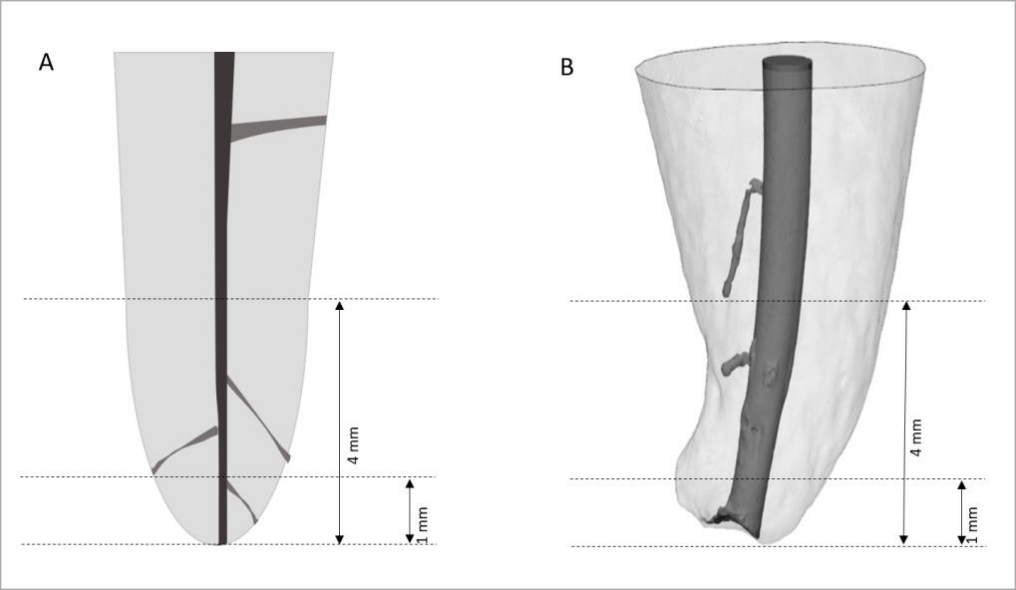
Representative images of the study methodology: single-rooted premolars with the main canal and its ramifications in 0-4 mm and 0-1 mm from the apical foramen.

For post-processing and image visualization the CTAn software v.1.18.8.0 (Bruker) was used to evaluate the volume of interest (VOI) of the filling materials in the apical main canal in two different ranges (0-4 mm and 0-1 mm) and the apical ramifications separately. Then, three VOI were evaluated according to the protocol of Huang et al. ^11^, as such to allow for a more accurate assessment of filling materials: VOI 1 - from the foramen up to 4 mm of the main canal in coronal direction (0-4 mm), VOI 2 - from the foramen up to 1 mm of the main canal in coronal direction (0-1 mm), and VOI 3 - root canal ramifications from the foramen up to 4 mm in coronal direction (figure 1).

A - Two-dimensional illustration. B - Three-dimensional reconstruction

### Statistical analysis

Statistical analysis was performed using JASP software (0.13 version, University of Amsterdam, Netherlands). Shapiro-Wilk test was used to verify the distribution of the sample and showed a non-normal distribution (p>0.05). The Mann-Whitney test was applied to compare the filled volume for both working lengths (G1 and G2) in the main canal apical range of 0-4 mm, 0-1 mm, and ramifications with a significance level of 5% (α ≤ 0.05).

## Results

**Figure 2:**
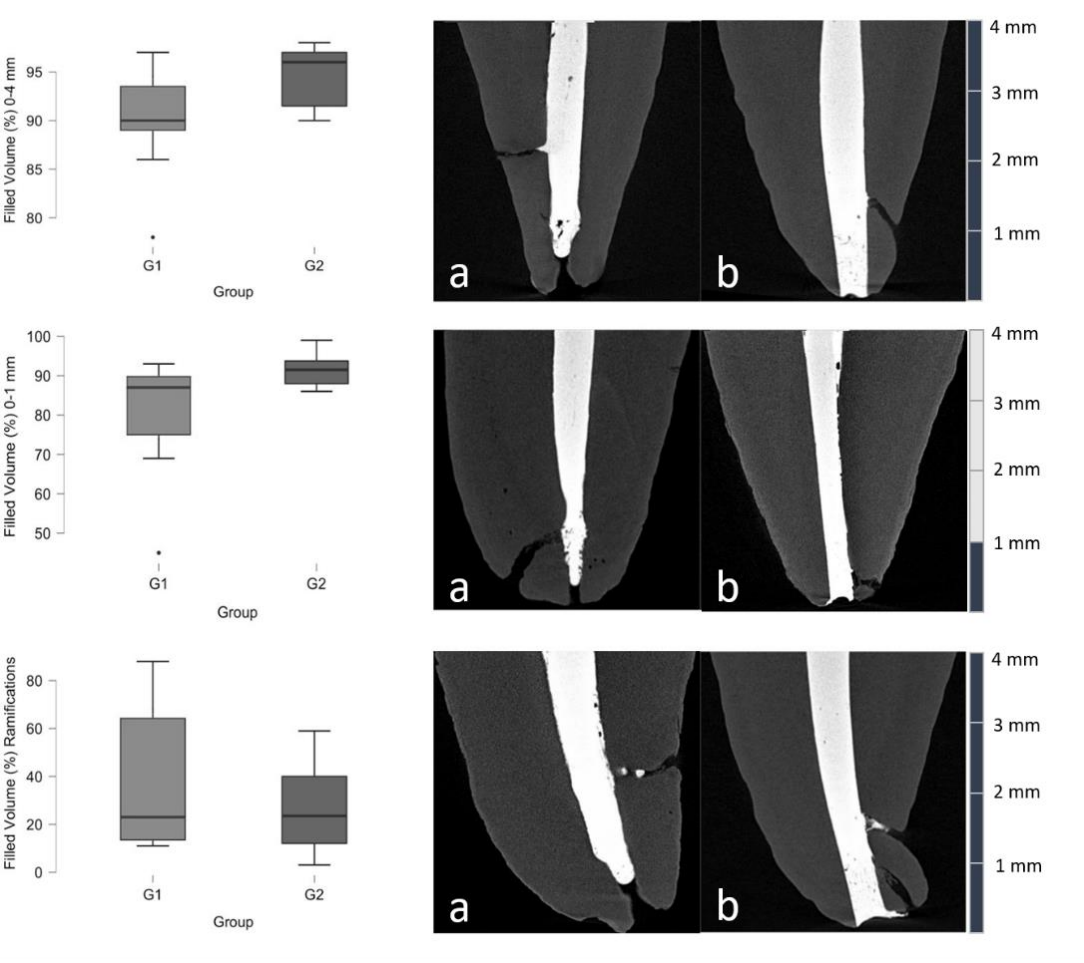
Percentage of filled volume for both working lengths in the main canal apical 0-4 mm, 0-1 mm, and ramifications, with representative reconstructed samples.

The percentage of filled volume in the main canal apical 0-4 mm range, 0-1 mm range and apical ramifications, with representative reconstructed samples are represented in Table 1 and Figure 2. G2 showed a median of 96% and 91.3% of filling in the main canal apical 0-4 mm and 0-1 mm ranges, respectively, whilst in G1 this percentage was significantly lower (p<0.05), showing 90% filling for the main canal apical 0-4 mm and 87% for the 0-1 mm range (Table 1). Regarding the filling of apical ramifications, in G2 23.5% of their volume was filled, and 23% was filled in G1, showing no statistical differences between groups (p>0.05).

**Table 1:** Percentage of filled volume for both working lengths (G1 and G2) in the main canal apical range of 0-4 mm, 0-1 mm, and ramifications.

	Filled volume (%) 0-4 mm		Filled volume (%) 0-1 mm		Filled volume (%) Ramifications	
	G1	G2	G1	G2	G1	G2
Median (Minimum-Maximum)	90 (78-97)	96 (90-98)	87 (45-93)	91.3 (86-99)	23 (11-88)	23.5 (3-59)
P-value of Shapiro-Wilk	0.187	0.017	0.014	0.686	0.026	0.589
P-value for the Mann-Whitney test	0.005		0.027		0.798	

## Discussion

The present study demonstrated that filling of main root canal system is influenced by instrumentation at different working lengths, accepting the hypothesis. Instrumentation at the apical foramen resulted in a larger volume of the main canal filled, in both apical 0-4 mm and 0-1 mm ranges (p<0.05), when compared to instrumentation 1 mm short. This finding suggests that instrumentation throughout the entire extent of root canal favors sealers’ penetration in all extension of the main canal. On the other hand, sealing of ramifications at 0-4 mm was not significantly affected by the working length. This information is particularly relevant because gaps and voids in the root canal space have the potential to work as a microbial reservoir, which may lead to treatment failure, hence, disinfection and sealing of the entire root canal must be bear in mind during endodontic therapy to prevent an inflammatory process ^6^ ^,^ ^15^ ^,^ ^16^.

A previous study has shown that the apical third is more likely to have several ramifications of the main pulpal canal ^2^. Moreover, the apical foramen and ramifications in the apical third are the connection between the main canal and the periodontal ligament space and may equally serve as a pathway for bacteria causing periapical disease ^1^. Because of the greater clinical impact of those anatomical structures, and aiming to select a more standardized sample, the present analysis was focused on the apical third.

A previous work evaluated the influence of different working lengths (1 mm short of the foramen; at the foramen; and 1 mm over the foramen) on filling of the apical foramen, and concluded that instrumentation 1 mm short resulted in more unfilled areas ^17^. Those results are in accordance with the present study; additionally, our study shows relevance as the entire volume of the apical region of root canals, as its ramifications, were evaluated. The underfilled spaces in main canals instrumented 1 mm short of the foramen may be explained by the preparation size of the apical region, possibly because of a smaller space available for sealers’ penetration in all its extension. In the present study, the working length was the only variable; however, since a finishing file size 40.06 was used at the foramen level in G2, naturally this group had apical regions with larger volumes, which may have favored sealers’ penetration.

Concerning the root canal ramifications, in the present, they were poorly filled by the sealer in both groups. Despite connecting the main canal to the periodontal ligament, the role of ramifications on treatment outcome is still a matter of debate; while some studies agree that ramifications are not a reason for treatment failure, others believe that because they are unlikely to be cleaned by endodontic instruments, biofilm growth and treatment failure are favored ^10^ ^,^ ^13^ ^,^ ^16^ ^,^ ^18^. Ricucci and Siqueira ^18^ observed that endodontic materials never completely filled the apical ramifications due to their small diameters. Similarly, in the present study, this may have interfered with the sealer capacity to penetrate those spaces. Yet, another possible reason for unfilled ramifications is the presence of debris, observed during imaging analysis. Even though debris formation is invariably present during instrumentation, some clinical conducts could be followed to reduce their formation and intensify the penetration of sealer in areas of anatomic complexity ^19^; examples may include agitation of irrigating solutions and obturation using thermoplasticized techniques ^20^ ^,^ ^21^. In addition, recent studies have recommended the use of ultrasonic activation of sealers to favor better quality of root canal filling and to increase intratubular penetration ^22^ ^,^ ^23^. These procedures were not included in the present study, since our aim was to evaluate the influence of the instrumentation length on root canal filling in the apical region and without any additional intervention. Further clinical studies including the aforementioned procedures are recommended for investigating the performance of different sealer materials, especially regarding apical ramifications, crossing clinical information with those obtained from *ex vivo* studies.

The visualization of fine details in this study can be attributed to nano-CT images, an emerging technology with the potential to provide new insights over the influence of root canal anatomy on endodontic therapy ^12^. Many studies in endodontics have used micro-CT as the reference standard, however, nano-CT presents a technical advancement of this established technology, with higher-definition images, excellent contrast sensitivity, smaller voxel sizes, and significantly lower scanning time ^24^. In a direct comparison between micro-CT and nano-CT, both methods revealed similar sensitivity, but images with better resolution and more details for nano-CT ^25^. Mavridou et al. ^24^ observed that nanoCT images (voxel size of 7 μm) were more accurate than those of CBCT and micro-CT (15 μm), in investigating details of external cervical resorption. Huang et al. ^11^ compared micro-CT and nano-CT for the quantitative analysis of sealer filling quality and observed that nano-CT has greater ability of distinguishing internal porosity, therefore suggesting its use for filling materials analysis. Moreover, the evaluation of high-density materials using nano-CT is advantageous because of the absence or minimal artifact formation, due to the large number of projection images acquired, higher sensitivity, and software that allowed the control of the beam hardening phenomenon ^14^. The use of this recent tomographic method will allow better observation of minor details inside the root canal system, such as voids and debris, guiding future studies on establishing a treatment protocol that improves cleaning and filling of root canal system.

Despite its advantages, nano-CT cannot be reproduced in an *in vivo* scenario and, therefore, is not feasible for clinical evaluation of root canals anatomy due to the inherent high energy exposures parameters, which generate more radiation dose. A limitation of this*ex vivo*study was to obtain standardized tooth samples, with the same amount of ramifications and volumes of the main canals. In order to overcome this limitation, meanwhile preserving the tooth anatomy, single-rooted premolars with one main canal and up to 3 apical ramifications were carefully selected. Further studies should investigate different dental groups and the performance of other filling materials using various treatment strategies.

In conclusion, the root canals shaped at apical foramen exhibited increased filling volume of the main canal. However, neither of both working lengths influenced filling of the apical ramifications.
